# Hydrogen Breath Tests: Are They Really Useful in the Nutritional Management of Digestive Disease?

**DOI:** 10.3390/nu13030974

**Published:** 2021-03-17

**Authors:** Paolo Usai-Satta, Francesco Oppia, Mariantonia Lai, Francesco Cabras

**Affiliations:** 1 Gastroenterology Unit, Brotzu Hospital, 09121 Cagliari, Italy; f.oppia@tiscali.it (F.O.); francescocabras@aob.it (F.C.); 2 Gastroenterology Unit, University of Cagliari, 09042 Monserrato, Italy; toninalai@medicina.unica.it

**Keywords:** hydrogen breath testing, lactose malabsorption and intolerance, fructose and sorbitol malabsorption, FODMAP, nutritional management

## Abstract

Background: Carbohydrate malabsorption is a frequent digestive problem associated with abdominal pain, bloating and diarrhea. Hydrogen breath testing (BT) represents the most reliable and validated diagnostic technique. The aim of this manuscript was to clarify the usefulness of BTs in the nutritional management of these disorders. Methods: A literature search for BT related to carbohydrate malabsorption was carried out using the online databases of Pubmed, Medline and Cochrane. Results: Lactose BT showed good sensitivity and optimal specificity for lactose malabsorption. However, an accurate diagnosis of lactose intolerance should require blind lactose challenge although this method is difficult to utilize in clinical practice. Regarding dose-depending fructose and sorbitol malabsorption, BTs could not add diagnostic advantage compared with a direct dietary intervention. In addition, carbohydrates are fundamental components of fermentable oligo-, di- and monosaccharides and polyols (FODMAPs). Before starting a low FODMAP diet, lactose BT should be suggested in a population with low prevalence of hypolactasia. Conclusions: BTs represent a valid and noninvasive technique in many digestive conditions. Regarding the management of carbohydrate intolerance, lactose BT can be recommended with some limitations. No sufficient evidence is available about the usefulness of BTs for other sugars in clinical practice.

## 1. Introduction

Carbohydrate malabsorption is a frequent clinical condition which may be responsible for digestive symptoms such as abdominal pain, bloating and diarrhea both in adults and in children [1]. A large number of studies have been dedicated to lactose malabsorption and intolerance, although fructose and sorbitol also merit clinical attention in clinical practice [2,3,4,5]. Lactose, fructose and sorbitol are also relevant components of fermentable oligo-, di- and monosaccharides and polyols, the so-called FODMAPs [6]. A low FODMAP diet is considered an important dietary intervention to ameliorate symptoms in patients with irritable bowel syndrome (IBS). The mechanism by which these malabsorbed sugars may cause symptoms is related to an osmotic action that increases the volume of intestinal contents and leads the undigested carbohydrates to be fermented by the colonic flora. Different methods have been used to perform the diagnosis of sugar malabsorption. Hydrogen (and/or methane) breath testing (BT) is generally considered the most reliable and validated diagnostic technique [7,8]. On the other hand, the usefulness of BT in proving the responsibility of sugar malabsorption for intolerance symptoms remains controversial. Nutritional implications are important in this clinical setting and the complete exclusion of these sugars from the diet is not always necessary and can potentially cause serious nutritional consequences. The aim of this review was to clarify if hydrogen BT can be useful to guide nutritional intervention in the presence of digestive intolerances. 

A review of literature on BT related to sugar malabsorption was conducted using the online databases of Pubmed, Medline and Cochrane. In particular, we searched for the diagnostic usefulness and validity of BT in the nutritional management of carbohydrate malabsorption and intolerance. The most relevant original research, review, systematic review and meta-analysis and eventually books were included in the search. The search was performed in various combinations with the Boolean operators “and”, “or” and “not”, selecting articles published in English.

## 2. Breath Testing

### 2.1. Background

The methodological principles of BT are based on the concept that part of the gas produced by colonic bacteria fermentation diffuses into the blood, is rapidly excreted by breath and can be consequently measured and quantified by dedicated instruments [9,10,11].

Regarding the intraluminal production, CO_2_, H_2_ and CH_4_ represent the predominant gases generated in the entire gastrointestinal tract.

In fasting conditions, H_2_ production is normally low, but after ingestion of fermentable and undigested substrates, intraluminal bacteria produce relevant amounts of H_2_. In particular, when carbohydrate absorption is impaired, high amounts of undigested material reach the colon and become available for bacterial fermentation, producing gases (CO_2_, H_2_, CH_4_) and organic acids, such as lactic acid and short-chain fatty acid [12].

The gases not utilized by bacteria are absorbed and then excreted in breath. About 15–20% of H_2_ released in the colon is excreted by the lungs [13].

The so-called low H_2_ producers fail to produce adequate amounts of breath hydrogen following sugar malabsorption. The presumed prevalence of low H_2_ producers is about 20% of the general population. The reduced production of hydrogen should be related to increased hydrogen consumption rather than reduced production. About 30% of the adult population (the so-called CH_4_ producers) presents high concentrations of methanogenic flora, able to consume large quantities of hydrogen to produce methane [12]. On the other hand, the optimal criterion to define excessive methane production is not clear. In patients who are low H_2_ producers, methane BT could represent an alternative method to hydrogen BT. Unfortunately, not all low H_2_ producers are methane producers and the concomitant measurement of H_2_ and CH_4_ could not add diagnostic advantage. In fact, no validation studies are available to suggest an optimal cut-off and a defined procedure to perform CH_4_ BT. Finally, further factors may affect hydrogen (and methane) production such as bowel motility, antibiotics and laxatives [14].

### 2.2. Methodological Aspects

In clinical practice, breath measurement of gases may be performed by dedicated gas chromatographs and more recently by portable devices. Dedicated stationary gas chromatographs measure hydrogen alone or in combination with methane.

They are relatively expensive and represent the gold standard for hydrogen measurement in breath condition. As an alternative, portable instruments are available and have the characteristics of being more practical and less expensive. They use a different methodology based on electrochemical cells, demonstrating good accuracy and reproducibility [14].

Due to their low cost, simplicity and reproducibility, BTs are very popular and widely performed. On the other hand, the lack of standardization regarding the indications, preparation, performance and interpretation has led to considerable heterogeneity between different centers and practitioners. For these reasons, several attempts have been made to recommend standardized procedures.

Currently, there are two main consensus documents available regarding BT, published by the Italian Consensus Conference Working Group and by the North American consensus group in 2009 and 2017, respectively [15,16].

Presently, in clinical practice, BT is performed with various substrates, such as lactose, fructose and sorbitol for carbohydrate malabsorption; glucose (and lactulose with conflicting results) for small intestinal bacterial overgrowth; and lactulose for orocecal transit.

According to these consensus documents, the accuracy of H_2_ BTs must be based on a correct test protocol. In particular, devices for breath sampling comprise the Y-piece device, and the two-bag system and gas measurement should be performed within 6 h from the collection.

Laxatives and antibiotics must be avoided for a period of 4 weeks before the test. Cigarette smoking modifies breath hydrogen excretion and must be avoided before and [17] during the test.

A restricted diet, free of non-absorbable carbohydrates the evening before, as well as an overnight fast must be adopted.

Mouth washing with chlorexidine solution before the substrate administration prevents oral fermentation by bacterial flora of the oral cavity.

Finally, physical exercise must be limited during the test because of the risk of reduced breath hydrogen secretion by hyperventilation. Table 1 summarizes the suggested protocol before performing BT.

## 3. Lactose Malabsorption and Intolerance

### 3.1. Background

Lactose is a disaccharide composed of glucose and galactose bound in a ß-glycosidic linkage. It is the primary carbohydrate present in milk and dairy products. Absorption of lactose requires lactase-phlorizin hydrolase (LPH) activity in the small intestinal brush border. Lactose malabsorption (LM) is directly caused by an LPH deficiency, the so-called hypolactasia [2,3,4]. Hypolactasia comprises three distinct forms: congenital, primary and secondary.

Congenital lactase deficiency is an extremely rare lifelong disorder characterized by failure to thrive and severe infantile diarrhea from the first exposure to breast milk. Congenital hypolactasia is caused by a single autosomal recessive disorder characterized by five distinct mutations in the coding region of the lactase gene [18].

Primary “adult-type” hypolactasia is the most frequent condition related to LM occurring in a large proportion of individuals. It is an autosomal recessive condition resulting from the physiological decline of lactase enzyme activity in the intestinal cells. A single nucleotide polymorphism, C⁄T-13910, 14 kb upstream the lactase gene, has been correlated with lactase persistence or non-persistence in several populations [19]. The onset of “adult-type” hypolactasia is correlated to age: lactase activity is highest at birth and declines after weaning up to 8–12 years of age [20]. The frequency of this condition varies across world countries with reported lower prevalence in Northern Europe (<5%), compared to Southern Europe (70–90%) and Southeast Asia (almost 100%).

Finally, secondary hypolactasia is due to several diseases such as celiac disease, gastroenteritis and Crohn’s disease which may lead to a transient lactase deficiency.

LM can be responsible for several symptoms such as diarrhea, bloating, flatus and abdominal pain, namely lactose intolerance (LI). However, hypolactasia does not necessarily result in the development of intolerance symptoms [21,22]. The presence of digestive symptoms after ingestion of lactose is a function of many variables, including the amount of lactose, foods co-ingested with lactose, the lactose fermentation pathways of the colonic flora and the visceral sensitivity. Many subjects diagnose themselves as being lactose intolerant. However, these self-identified lactose intolerant individuals may actually be lactase persistent [21]. In addition, some hypolactasic patients may mistakenly ascribe the symptoms of IBS to LI.

### 3.2. Role of BT in the Management of LM/LI

The classic diagnostic gold standard for LM is represented by lactose assay after jejunal biopsy [2,3,4,9]. However, jejunal biopsy is too invasive for the diagnosis and its results may be influenced by the irregular dissemination of lactase activity throughout the small intestine mucosa.

Although it is an indirect test for LM, lactose BT is commonly considered the most reliable, non-invasive and inexpensive technique.

According to Italian and North American consensus documents [15,16], the following recommendations have been suggested on how to perform lactose BT: a dosage of 25 g lactose, a test duration of 4 h (3 h for pediatric use), sample intervals of 30 min and a cut-off value of 20 ppm above baseline to confirm LM. According to validation studies, lactose BT shows good sensitivity (mean value of 77.5%) and excellent specificity (mean value of 97.6%) [23]. Some risk of finding false negative results may be due to the inability of colonic flora to produce H_2_ after ingestion of lactose or after a recent administration of antibiotics. False positive BTs are less frequent and are mainly produced because of small bowel bacterial overgrowth [8,22].

More recently, a genetic test based on the C/T-13910 polymorphism, associated with the lactase persistence/non-persistence, has been proposed as a new diagnostic instrument for hypolactasia [24]. Several studies have demonstrated an excellent correlation between BT and the genetic test [25,26]. In subjects with negative lactose BT, the genetic test provides an unambiguous result, permitting the exclusion of false negative results. In addition, secondary causes of hypolactasia may be suspected in subjects with a positive BT and a negative genetic test [23,27]. In pediatric populations, the genetic test is not recommended before 8–12 years of age because the lactase decline and the onset of “adult-type” hypolactasia should be evident from this age onwards [20]. In these younger subjects, BT represents the only test useful to detect secondary LM.

The recording of four symptoms (abdominal pain, bloating, flatulence and diarrhea) during the BT and 8 h after, following lactose challenge, is commonly considered the most reliable diagnostic method to demonstrate LI in clinical practice [8].

A lactose-free diet for one or two weeks followed by a reintroduction of lactose can also be considered a simple and practical diagnostic method. On the other hand, only some patients with positive BT report symptoms after lactose challenge and conversely some lactose absorbers diagnose themselves as being lactose intolerant [21,22].

Recently, some authors have hypothesized that the psychological profile can influence the symptoms of LI when a more physiological dose of 15 g of lactose was administered during BT [28]. A multivariate logistic analysis showed that a high somatization t-score was significantly associated with LI. This study concludes that digestive symptoms attributed to LI could not depend on the presence of LM.

For these reasons, a blind lactose challenge should be the recommended method to objectively demonstrate LI, although this approach is difficult to realize in clinical practice [29].

In the presence of LM/LI, a therapeutic strategy consists of a lactose-restricted diet and the use of milk and dairy products in which the lactose has been prehydrolyzed, avoiding the nutritional disadvantages of reduced calcium and vitamin intake [23]. In fact, a complete exclusion of lactose from the diet is not necessary and can potentially cause serious nutritional consequences. In addition, various lactase supplements are commercially available [23].

## 4. Fructose Malabsorption

### 4.1. Background

Fructose exists mainly in three forms: monosaccharide; disaccharide, the sucrose, composed of fructose and glucose; and polymerized forms such as oligosaccharides (fructans) and polysaccharides [30]. Fructose is naturally present in fruits and vegetables, such as apples, peaches and prunes. It is also produced enzymatically from corn as high-fructose corn syrup and this form of fructose is commonly used in many food sweeteners.

Unlike lactose and sucrose, fructose is not digested by an enzymatic action but is absorbed by a dose- and concentration-related transportation system via the brush border of the small bowel [5].

The mechanisms by which fructose is absorbed from the small intestine involve the glucose transporter (GLUT) pathways. GLUT5 represents the main intestinal fructose transporter, but other intestinal transporters could be involved. GLUT2 and SGLT4 are also present in the small bowel and might play a role in fructose absorption. In particular, GLUT5 or GLUT2 expression could be reduced in fructose malabsorption (FM) [30,31]. Glucose stimulates fructose absorption in a dose-dependent manner, and malabsorption can occur when fructose is present in excess of glucose. On the other hand, whether fructose absorption can be enhanced by increased GLUT5 expression due to a fructose-rich diet in humans is not yet definitely clarified.

FM is a highly prevalent disorder and a frequent gastrointestinal diagnosis. It ranges from 38 to 80%, depending on the fructose dose.

Patients with FM experience symptoms of bloating, abdominal pain and diarrhea [5]. In these patients, the absorption of fructose can be impaired by an ineffective monosaccharide transportation system and this leads to a higher concentration of fructose in the colon. When fructose is ingested in large amounts, the capacity of the gut to absorb fructose can be easily overwhelmed, thus leading to FM. As a consequence, fructose is metabolized by colonic bacteria to hydrogen, methane and short-chain fatty acids, causing digestive symptoms. Apart from primary FM, celiac disease, Crohn’s disease or acute bowel inflammations can cause secondary and transient forms of FM. Finally, high fructose consumption can trigger symptoms in patients with IBS [31].

### 4.2. Role of BT in the Management of Fructose Malabsorption

Hydrogen fructose BT is commonly considered the most reliable and simple technique to detect FM. The recent North American consensus conference [16] has suggested performing fructose BT with a dosage of 25 g fructose, a duration of at least 3 h and a cut-off of 20 ppm of hydrogen from baseline to consider a positive result. Unlike the North American document, the Italian consensus conference working group in 2009 [15] did not recommend BT for FM in clinical practice. No gold standard available for fructose BT and no significant published validation studies led to this conclusion. In addition, the appropriate fructose dose for this test is still controversial [32,33]. In a classic study [34], after ingestion of 50 g fructose, the majority of patients showed a positive BT and about 25% reported abdominal symptoms during the test period. Using 25 g, only 11% of subjects showed FM, of which only one patient had symptoms. A double blind study [35] performed in 20 healthy subjects, showed 80% of positive tests and 55% of abdominal symptoms after a dosage of 50 g fructose. In other words, 50 g fructose exceeded the intestinal absorption capacity and can provoke symptoms even in healthy subjects. Thus, 15–25 g can be a more valuable dosage to perform BT but it is hardly possible to define the right dose able to discriminate between normal and abnormal absorption. In a recent study [36], H_2_ fructose BT had no positive predictive value for a fructose-free diet. In addition, fructose BT had poor reproducibility and low predictive value in 21 patients with functional bowel disorders [37]. Similarly to lactose intolerance, only a blind fructose challenge could objectively demonstrate a relationship between FM and digestive symptoms. In conclusion, a restricted fructose diet could be suggested in clinical practice after a dietary suspect for FM without performing BT.

## 5. Sorbitol Malabsorption

### 5.1. Background

Sorbitol is a sugar alcohol naturally present in fruits and vegetables, such as apples, peaches and prunes. It is also produced synthetically for commercial use and it can be found in sweets, chewing gum, dietetic foods and drugs. Sorbitol is normally poorly absorbed by the small bowel. Its intestinal absorption is not mediated by an enzymatic action but occurs by a dose- and concentration-related diffusion mechanism via the brush border of intestinal mucosa [5,38]. A specific transport system for sorbitol absorption has not been identified. However, the absorption capacities for sorbitol seem to correlate with those of fructose. Fructose and sorbitol could have a common transporter and sorbitol could interfere with the fructose absorption.

Sorbitol malabsorption is considered a very frequent problem in the general population. Test solutions containing 10 g and 20 g resulted in 90% and 100%, respectively, of healthy volunteers showing malabsorption [39]. When sorbitol is ingested in large amounts, the capacity of the gut to absorb it can be compromised, leading the sorbitol to be metabolized by colonic bacteria to hydrogen, methane and short-chain fatty acids and provoking digestive symptoms. Similarly to fructose, patients with sorbitol malabsorption can suffer from bloating, abdominal pain and diarrhea.

### 5.2. Role of BT in the Management of Sorbitol Malabsorption

The diagnosis of sorbitol malabsorption is commonly obtained by hydrogen BT. However, no gold standard and no published validation studies are available for sorbitol BT. In addition, the dosage of sorbitol for BT is not standardized [40]. Generally, BT is performed after an administration of 10–20 g sorbitol, breath samples are collected every 30 min for 4 h and hydrogen elevation higher than 20 ppm over the baseline is considered to define malabsorption [38].

Due to this methodological weakness, the Italian consensus conference working group [15] did not recommend sorbitol BT in clinical practice.

Some studies have suggested utilizing sorbitol BT with smaller dosage in untreated celiac disease. The ingestion of 5 g sorbitol provoked a highly significant increase in H_2_ excretion as compared with healthy subjects [39]. Positive 5 g sorbitol BT seems to correlate with histologic damage and be predictive of villous atrophy [41]. Furthermore, BT could also be useful in the follow-up of celiac patients on gluten-free diet [42]. On the other hand, sorbitol BT has not been generally adopted for celiac disease work-up in clinical practice.

In conclusion, a reduced sorbitol diet should be suggested in patients following a diet rich in sorbitol related to digestive symptoms. According to several pieces of evidence, BT does not add a diagnostic advantage in patients with suspected sorbitol malabsorption.

## 6. FODMAPs Intolerance

### 6.1. Background

FODMAP is an acronym coined to describe foods with highly fermentable oligo-, di- and monosaccharides as well as polyols. Based on recent evidence, FODMAPs can trigger and exacerbate symptoms in patients with IBS and functional gastrointestinal disorders [6].

FODMAPs are osmotically active carbohydrates that are poorly absorbed and rapidly fermented by gut bacteria. The mechanism of poor absorption is thought to be related to a low-capacity transport mechanism across the epithelium (fructose), reduced activity of brush border hydrolases (lactose), lack of hydrolases (fructans, galactans) or molecules being too large for absorption (polyols) [6]. Increased intraluminal water volume, due to osmotic activity and gas production from their fermentation, causes bowel luminal distension and provokes digestive symptoms.

Therefore, a low FODMAP diet (LFD) has the potential to ameliorate digestive symptoms in IBS and functional gastrointestinal disorders [43].

The response to an LFD may be related to patient demographics, microbiome composition and metabolism, diet adherence and IBS subtype.

Restriction of individual FODMAPs (e.g., lactose, cereals or vegetables) has been suggested in the management of IBS, but an LFD is a more general restriction. In fact, all FODMAPS are potential triggers of symptoms but not all actually induce abdominal symptoms in IBS. Therefore, a personalized, long-term FODMAP restriction (adapted LFD) can be suggested. Another crucial issue is the reintroduction phase which is necessary to determine individual tolerance to a specific food [44].

A recent meta-analysis [45] showed that IBS patients receiving an LFD experienced a statistically significant pain and bloating reduction compared with those receiving a traditional diet.

However, a blind challenge method should be preferred to achieve objective results, but this approach is particularly difficult to realize considering too many diet components and variables.

In addition, some potential problems about LFD should be highlighted. It may be more expensive than a habitual diet; it may reduce beneficial species of gut microbiota; it could decrease the fiber intake and could be constipating; it could be nutritionally dangerous (e.g., excessive weight loss; reduction of serum iron, calcium and vitamins); and it could be effective only in the short term [44,46].

In any case, a strict long-term LFD cannot be necessary and a careful follow-up of the patients on an adapted and personalized LFD is mandatory.

### 6.2. Role of BT in the Management of FODMAP Diet

The original protocol of an LFD provided for a diagnostic work-up [6]. A preliminary study by lactose, fructose and sorbitol BTs was suggested to avoid carbohydrate-restricted diet in absence of lactose, fructose and/or sorbitol malabsorption. On the other hand, performing BTs is not considered absolutely necessary before starting this diet.

The selection criteria for the LFD have evolved from patients with malabsorption demonstrated by BTs, to patients with symptoms provoked by high doses of sugars, to IBS patients without further testing. In fact, BTs are not currently performed and an LFD is in any case initiated excluding lactose, fructose and sorbitol [47].

For research purposes, BTs have also been used to demonstrate a reduction in breath hydrogen amount during and after an LFD [48].

Regardless of specific sugar malabsorption or IBS, performing lactose BT before an LFD could be especially useful in populations with low prevalence of hypolactasia, avoiding the exclusion of lactose in lactase persistent patients.

Unlike lactose, the malabsorption of fructose and sorbitol is dose-dependent and BTs for these sugars cannot add diagnostic advantage to guide nutritional intervention related to an LFD.

## 7. BTs and Other Carbohydrates

Trehalose is a disaccharide found in mushrooms, algae and insect hemolymph. Trehalase is a brush border beta-galactosidase which hydrolyzes the trehalose to two glucose molecules for absorption [40]. To our knowledge, only a single study [49] is available about trehalose malabsorption and hydrogen BT. Intolerant subjects were best differentiated from tolerant subjects by changes in breath hydrogen amounts.

Maltitol is a polyol used as a sweetener in several confectionery products [50]. In particular, it is an excellent substitute for sucrose in products such as non-sugar chocolate. Maltitol is also a component of FODMAP diet. The ingestion of maltitol is associated with increased levels of colonic fermentation and with dose-dependent digestive symptoms including abdominal pain, flatus and borborygmi. In a study published in 1998 [51], chocolate containing 40 g maltitol caused mild borborygmi and flatus but no diarrhea. An increased breath H_2_ response was present, indicating colonic fermentation of this polyol.

Sucrose is a disaccharide hydrolyzed to glucose and fructose by sucrase-isomaltase enzyme. Sucrase-isomaltase deficiency is an autosomal dominant disorder causing malabsorption of sucrose and digestive symptoms such as diarrhea, abdominal pain and bloating. According to recent evidence, genetic variants for this reduced enzymatic activity can be associated with IBS [52,53].

Hydrogen sucrose BT has been used to demonstrate sucrose malabsorption, particularly in pediatric populations [54,55]. More recently, sucrose BT (with stable isotope of carbon, C13) has been proposed as a biomarker of small bowel mucosal damage [56].

Nevertheless, no sufficient evidence is available to recommend BTs related to these carbohydrates in clinical practice.

## 8. Future Outlook

BTs are widely performed but the lack of standardization regarding the indications, preparation and interpretation of results has led to considerable heterogeneity between different centers. For these reasons, consensus documents have been developed to recommend a shared diagnostic protocol. However, several aspects remain partially unanswered, such as the real physiological dose of sugars, the real diagnostic usefulness of methane or other gases, the determination of a specific and validated protocol in pediatric age, the influence of age, gender, ethnicity and race, the role of microbiota and the effect of diet and pre-probiotics on hydrogen production. In any case, there are many challenges that may suggest further studies. In particular, no sufficient data are available about other diet substrates such as inulin, fructans and galactans or volatile organic compounds (VOCs). A better knowledge of gas metabolism related to gut microbiota and diet compounds could improve clinical management of gas-related disorders, IBS and gastrointestinal functional disorders and guide a personalized dietary intervention.

## 9. Conclusions

BTs represent a valid and non-invasive diagnostic technique in many digestive conditions. Consensus documents are available in order to recommend standardized BT procedures. Among various clinical indications, carbohydrate malabsorption and intolerance represent the most frequent clinical use for BTs. Table 2 summarizes diagnostic strengths and weaknesses of the more common hydrogen BTs for carbohydrates. Currently, lactose BT can be recommended for lactose malabsorption alone or related to an LFD. A genetic test for lactase non-persistence may complement the lactose BT in several aspects, improving the diagnosis of “adult-type” hypolactasia. Unlike lactose, BTs for other sugars, such as fructose and sorbitol, could not add diagnostic advantage to guide nutritional management of these carbohydrates compared with a direct dietary intervention. In any case, a blind sugar challenge remains the most valid technique to objectively demonstrate a clinical intolerance to carbohydrates, although this method is difficult to utilize in clinical practice.

## Figures and Tables

**Table 1:** Suggested protocol before performing breath testing.

Laxatives and antibiotics avoided for 4 weeks before the test
Smoking avoided before and during the test
Restricted carbohydrate diet the day before and overnight fast before the test
Mouth washing with chlorexidine before sugar administration
Physical exercise limited during the test

**Table 2:** Breath tests for sugar malabsorption: diagnostic strengths and weaknesses.

Breath Testing	Dosage	Strength	Weakness	Recommendations
Lactose	25 g	Simple and non-invasive; validated for LM	Poor evidence for lactose intolerance	Recommended for LM and before LFD
Fructose	25 g	Simple and non-invasive	Not validated, not gold standard	Not recommended for FM and before LFD
Sorbitol	10–20 g	Simple and non-invasive	Not validated, not gold standard	Not recommended for SM and before LFD
Trehalose	Not defined	Simple and non-invasive	Not validated, not gold standard	Not recommended
Maltitol	Not defined	Simple and non-invasive	Not validated, not gold standard	Not recommended
Sucrose	Not defined	Simple and non-invasive	Not validated, not gold standard	Not recommended

Notes: LM lactose malabsorption; FM-SM fructose-sorbitol malabsorption; LFD low FODMAP diet.
